# The Effects of Home Automation on Personal and Social Autonomies in Spinal Cord Injury Patients: A Pilot Study

**DOI:** 10.3390/jcm13051275

**Published:** 2024-02-23

**Authors:** Giuseppa Maresca, Desirèe Latella, Caterina Formica, Isabella Veneziani, Augusto Ielo, Angelo Quartarone, Rocco Salvatore Calabrò, Maria Cristina De Cola

**Affiliations:** 1 IRCCS Centro Neurolesi “Bonino-Pulejo”, S.S. 113 Via Palermo C. da Casazza, 98124 Messina, Italy; giusy.maresca@irccsme.it (G.M.); desiree.latella@irccsme.it (D.L.); augusto.ielo@irccsme.it (A.I.); angelo.quartarone@irccsme.it (A.Q.); roccos.calabro@irccsme.it (R.S.C.); mariacristina.decola@irccsme.it (M.C.D.C.); 2 Department of Nervous System and Behavioural Sciences, Psychology Section, University of Pavia, Piazza Botta 11, 27100 Pavia, Italy

**Keywords:** spinal cord injury, neurorehabilitation, home automation, activities of daily living, quality of life

## Abstract

*Background:* Spinal cord injury (SCI) is a severe and progressive neurological condition caused by trauma to the nervous system, resulting in lifelong disability and severe comorbidities. This condition imposes serious limitations on everyday life, interfering with patients’ social lives and compromising their quality of life, psychological well-being, and daily living activities. Rehabilitation is essential to helping SCI patients gain more independence in their daily routines. Home automation (HA) systems provide personalized support to users, allowing them to manage various aspects of their living environment, promoting independence and well-being. This study aims to demonstrate the efficacy of an HA system in enhancing personal and social autonomies in SCI patients, resulting in improved cognitive function and reduced anxiety–depressive symptoms compared to traditional training. *Methods:* We enrolled 50 SCI patients undergoing neurorehabilitation at IRCCS Centro Neurolesi (Messina, Italy). These patients were randomly assigned to one of two groups: a control group (CG) and an experimental group (EG). The CG received traditional training, while the EG underwent HA training. We evaluated the patients before (T0) and after (T1) rehabilitation using various scales, including the Montreal Cognitive Assessment (MoCA), the Beck Depression Inventory (BDI), the Hamilton Rating Scale for Anxiety (HRS-A), the 12-Item Short-Form Survey (SF-12), the Functional Independence Measure (FIM), Activities of Daily Living (ADL), Instrumental Activities of Daily Living Scale (IADL), and the EQ-5D-5L. *Results:* The effect of the experimental treatment showed an improvement in all patients test scores in the EG, especially regarding cognitive functions, mood disorders, activities of daily living, and quality of life. *Conclusion:* Our findings suggest that HA may be effective in improving daily autonomy and, in turn, alleviating mood disorders and enhancing psychological well-being.

## 1. Introduction

A spinal cord injury (SCI) is a neurological condition that causes physical, psychological, and psychosocial disabilities. Damage to the spinal cord can happen due to primary or secondary injury. Primary cord injury can involve different mechanisms like impact with temporary compression of the cord, impact with ongoing compression, distraction injury, and direct laceration or transection. Secondary injury can occur due to various local or systemic processes such as hypotension, hypoxemia, hemorrhage, and cord edema [1]. This type of damage can cause lasting or temporary changes in bodily function, movement, strength, and sensation below the location of injury [1]. In Italy, there are an estimated 1014 new cases of SCI every two years, with the majority being caused by trauma (67.5%) [2]. Among them, almost 57% of spinal cord injuries are sustained due to hyperextension of the neck, which results in the compression of the cervical spinal cord. This physical trauma can lead to total or partial quadriplegia and impaired muscle tone [3]. The location and type of SCI determine different clinical conditions, functional outcomes, and rehabilitation progress [4]. SCI can cause severe disabilities, primarily involving motor function, but also sense–motor, genito-urinary, neuro-vegetative, or respiratory impairments. SCI is acute and unexpected and drastically alters the course of an individual’s life. Individuals with SCI have to go through the process of learning basic skills all over again (e.g., eating, bathing, dressing…). Patients with spinal cord injury (SCI) face various challenges depending on the site of injury. These challenges include neurogenic bowel and bladder issues [5], respiratory symptoms [6], cardiovascular complications [7], alterations in sexual functioning [8], and chronic and neuropathic pain [9]. Apart from these motor symptoms, SCI can also lead to altered social functioning [10], depressive symptoms [11], anxiety disorders [12], and cognitive difficulties [13,14]. These complications can significantly impact a patient’s quality of life (QoL) and psychological well-being [15]. The rehabilitation process for these patients requires a significant amount of time and resources. This includes the involvement of multidisciplinary teams and dedicated equipment to achieve the best rehabilitation outcomes. The expenses incurred due to inpatient rehabilitation constitute a substantial portion of the overall cost of patient management expenses, typically ranging from 60 to 90% [16]. For this reason, in the field of rehabilitation, it is crucial to provide patients with a top-notch rehabilitative process. The primary aim is to enable them to become as self-sufficient as possible while also developing the necessary skills for them to use permanent aids. New technologies have been increasingly utilized in recent years to enhance patients’ personal autonomy, primarily in their activities of daily living [17,18]. However, after hospital discharge, patients often struggle with day-to-day activities at home, which can lead to frustration, apathy, and depressive symptoms [19]. Environmental control systems, such as home automation systems, can be helpful in improving their functional abilities and overcoming physical limitations, by enabling users to manage different aspects of their living space, such as lighting, windows, entrances, blinds, AC, heating, and various appliances like washing machines, refrigerators, and stoves. The homes of the future will be equipped with advanced technology to offer network-based services that can benefit seniors and patients with disabilities, providing them with greater control over their living environment [20]. Home automation (HA) seamlessly integrates various systems and devices, allowing users to control and automate their homes remotely. The systems are designed with a user-centric approach, incorporating information and communication technology, automation, architecture, and ergonomics to provide personalized services [20]. In this way, HA creates a supportive environment that enables patients to regain control over their daily lives. The tools used in HA should be simple to use and adjustable according to the patient’s needs [21]. However, previous studies have mostly focused on improving functional motor impairment, neglecting the cognitive and behavioral outcomes [22]. In an earlier research work, we employed HA to assist clinical and rehabilitation processes. As a result, stroke [23] and Parkinson’s patients [24] experienced better cognitive and social abilities, along with enhanced functional autonomy. Recent technological advances, such as virtual reality and exoskeletons, have demonstrated the potential to improve the motor performance of SCI patients [25], as well as their psychosocial well-being and QoL [26]. The effectiveness of rehabilitation programs is also positively linked to the improvement of depressive and anxiety symptoms. Therefore, it is crucial to include interventions aimed at promoting psychological adjustment and coping in inpatient rehabilitation programs [27].

This study adopts a comprehensive approach to comparing two types of rehabilitation treatment for SCI patients: conventional training and advanced rehabilitation technology treatment using HA. The study aims to assess how HA can affect personal and social autonomies in individuals with SCI, leading to better cognitive functioning and reduced anxiety–depression symptoms.

## 2. Materials and Methods

This is an experimental study, conducted using a parallel, randomized, controlled, non-blinded trial design assessing the effectiveness of traditional rehabilitation therapy vs. HA. The randomization was performed using a single sequence of random assignments (random.org).

### 2.1. Study Population

Fifty patients affected by SCI (mean ± SD age: 50.2 ± 15.2 years; 58.0% male) were enrolled in this study and randomly assigned to the experimental (EG: *n* = 25) or control (CG: *n* = 25) groups. They all attended the HA laboratory located in the Neurorehabilitation Unit of the IRCCS Centro Neurolesi “Bonino-Pulejo” (Messina, Italy) between 2018 and 2020. Table 1 contains a more detailed description of the two groups. All the participants in this study provided written informed consent, in accordance with the guidelines outlined in the 1964 Helsinki Declaration. The experimental protocol was approved by the local committee of IRCCS Centro Neurolesi “Bonino Pulejo”, Messina (approval number IRCCS 31-18).

Inclusion criteria: (i) age ≥ 18 years; (ii) diagnosis of SCI according to the AIS (American Spinal Injury Association Impairment Scale) classification [28]; (iii) a stable SCI condition (i.e., at least 3 months after injury); and (iv) the ability to follow verbal instructions, with a Montreal Cognitive Assessment (MoCA) > 20.

Exclusion criteria: (i) severe bone disease such as osteoporosis (T score < −2.5); (ii) severe pain; (iii) spasms or spasticity (Modified Ashworth Scale > 3) despite specific drug therapy; (iv) history of psychiatric illness or medical comorbidities (e.g., psychosis, epilepsy, colostomy, unresolved venous thrombosis, uncontrolled autonomic dysreflexia, skin irritations/injuries, and cardio-respiratory failure) potentially interfering with training.

### 2.2. Interventions

All patients were assessed by a neurologist and a psychologist before (T0) and after the rehabilitation treatment (T1).

**Figure 1:**
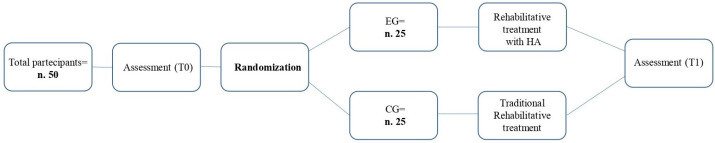
Method of assignment interventions.

Two distinct rehabilitation methods were implemented in the two separate groups: the GC underwent conventional training, while the EG was trained using advanced technologies available in the HA room (HA training) (Figure 1). Both treatments were administered by occupational therapists with expertise in rehabilitating patients with SCI using this technology (Table 2). The two treatments had the same duration and goal. During the training sessions, patients were guided through exercises that involved all limbs with a focus on promoting independence in activities of daily living. In the traditional training, patients were trained in small groups of 3–5 individuals, and the exercises included activities such as arranging beads, ball exercises, and manipulating objects like switches or zippers. Additionally, patients performed daily activities (e.g., cooking classes). On the other hand, the EG received training with the help of technological tools using HA. The HA training involved group activities with 3–5 patients per group, and the sessions took place in a dedicated HA room. The room had a range of tools that could be adjusted to meet the specific requirements of each patient, and it was provided by Allmobility Trading, Italy, while the table was supplied by Ergoswiss AG, Switzerland. The providers trained the therapists to use the equipment. In the HA room, patients had access to a centralized control system that allowed them to monitor and modify environmental parameters such as detecting smoke, water, or gas leakage [23,24]. The kitchen shelves and other storage areas were adjustable in terms of their size and depth, while the kitchen wall units, hood, and sink had axial automation. Furthermore, in the smart kitchen, plenty of utensils were available, including multi-purpose platters that ensured safe and effortless single-handed use, but also ball tools, good handles, and foldable cutlery. There was a toilet and shower in the bathroom that could be adjusted to different needs. Patients were trained and supported by the therapist in performing day-to-day tasks with HA, including cooking, dishwashing, oral hygiene, showering, and more. The training involved 24 sessions over a period of 8 weeks with 3 sessions per week, each lasting for approximately 60 min. The patients in both groups received the same amount of treatment.

### 2.3. Outcome Measures

The neuropsychological assessment included the following scales: the Montreal Cognitive Assessment (MoCA) to assess general cognitive status; the Beck Depression Inventory (BDI) [29] for assessing depression; the Hamilton Rating Scale for Anxiety (HRS-A) [30] to evaluate anxiety; the 12-Item Short-Form Survey (SF-12) [31] to evaluate perception of health status; the Functional Independence Measure (FIM) [32] to have a standard measure of disability; Activities of Daily Living (ADL) and Instrumental Activities of Daily Living Scale (IADL) [33] to evaluate the functional and instrumental autonomy of daily life; and, finally, the EQ-5D-5L to measure QoL [34].

### 2.4. Montreal Cognitive Assessment (MoCA)

The MoCA test evaluates all seven cognitive domains: memory, language, orientation, executive functions, praxis, visuospatial abilities, and attention. A score of ≤25 indicates impairment, with high test–retest (0.945) and inter-rater (0.999) reliability. Sensitivity and specificity are adequate at 95.3% and 84.5%, respectively [35].

### 2.5. Beck Depression Inventory (BDI)

The BDI measures depression severity, categorizing scores into minimal (0–13), mild (14–19), moderate (20–28), or severe (29–63) depression. The retest reliability ranges from 0.73 to 0.96, with significant diagnostic accuracy, exceeding 75% [36].

### 2.6. Hamilton Rating Scale for Anxiety (HRS-A)

The HRS-A assesses anxiety severity, with reliability coefficients ranging from 0.75 to 0.85 for global rating, psychic anxiety, and somatic anxiety. Some items exhibit lower reliability [37].

### 2.7. Activities of Daily Living (ADL) and Instrumental Activities of Daily Living Scale (IADL)

The ADL and IADL scales assess an individual’s ability to perform basic and complex daily functions. ADL items, such as eating, walking, toileting, bathing, grooming, and selecting clothes, require a forced-choice response. Scores range from 0 to 6, where lower values indicate greater disability [38]. Both the ADL and IADL scales have demonstrated good test–retest reliability (0.41–0.70) and high inter-rater reliability (0.85) [39].

### 2.8. 12-Item Short-Form Survey (SF-12)

The validity of the SF-12 was evaluated by comparing the physical and mental component scores of the survey across different groups of people. The Mann–Whitney and Kruskal–Wallis tests were used for this purpose. The SF-12 survey scores indicated that the physical and mental aspects of health had average scores of 49.6 (with a standard deviation of 9.0) and 51.9 (with a standard deviation of 8.6), respectively. Additionally, the reliability of the survey was confirmed using high Cronbach’s alpha coefficient (α = 0.836) [40].

### 2.9. Functional Independence Measure (FIM)

The FIM consists of 18 items assessing two domains: motor (13 items) and cognitive (5 items). Each item is scored on a 7-point scale, reflecting the level of assistance needed. The total score ranges from 18 to 126, indicating the degree of independence [41]. Studies confirm the FIM’s reliability and validity. The intra-class correlation coefficients (ICC) for the motor and cognitive domains are 0.96 and 0.91, respectively [42]. The construct validity is supported by variations in scores based on factors like age, comorbidity, and impairment severity [43]. The FIM demonstrates strong reliability, with median interrater, test–retest, and equivalence reliability values ranging from 0.92 to 0.95. Overall, the FIM is a consistent and robust tool for assessing functional independence [32].

### 2.10. EQ-5D-5L

The EQ-5D-5L’s high reliability and strong convergent validity are well established. When compared with its predecessor, the EQ-5D-3L, the 5L version demonstrated even stronger convergent validity coefficients, ranging from 0.51 to 0.75 (Spearman’s coefficients). Moreover, the intraclass correlation coefficients (ICCs) for the 5L items ranged from 0.61 (mobility) to 0.77 (anxiety/depression) [44].

### 2.11. Statistical Analysis

The data were analyzed using R version 4.2.2, considering a *p* < 0.05 as statistically significant. Since most of the target variables were not normally distributed, a non-parametric analysis was performed. Hence, the Mann–Whitney U test and Fisher’s Exact Test were used to compare the two groups at baseline/follow-up, when appropriate. We performed an analysis of covariance (ANCOVA) to assess whether the type of treatment influenced the clinical outcome, independently from the score difference at baseline. Notably, the model had the test score at T1 as the dependent variable, the categorical variable “Group” (1 = experimental; 0 = control) as the independent variable, and the test score at T0 as a covariate. We performed ANOVA to verify whether the model was significantly different from the one fitted with the interaction term “Group * test score at T0”. Finally, we used the Wilcoxon signed-rank test to compare each group between the baseline and the end of the study (intra-group analysis).

## 3. Results

**Figure 2:**
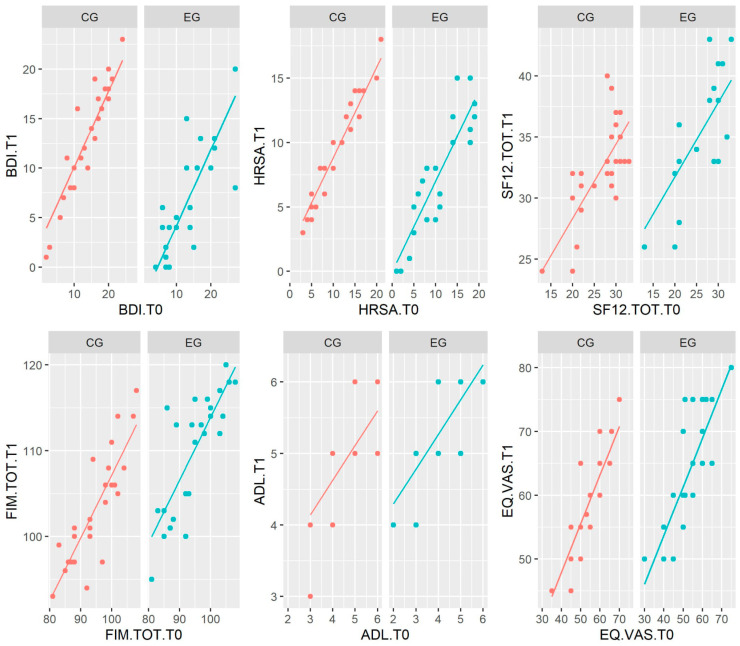
Plot of the predicted values from the covariate models for both groups (EG = experimental group; CG = control group), where the covariate is the test score at T0, and the outcome variable is the test score at T1.

No significant differences at baseline between the clinical assessment scores of the two groups were found, whereas at follow-up, the two groups differed in their MOCA scores (*p* < 0.01). The ANCOVA assumptions were always satisfied, except the one of homogeneity of variances in the MOCA scores. Since the interaction term “Group * test score at T0” was not significant, it was not considered in the ANCOVA model fitting, except for FIM.COG. The results reported in Table 3 show that the effect of the two treatments was significantly different for the BDI (t = −6.93; *p* < 0.001), HRSA (t = −4.03; *p* < 0.001), SF12.MH (t = 2.04; *p* = 0.047), SF12.PH (t = 2.96; *p* = 0.005), SF12.TOT (t = 3.88; *p* < 0.001), FIM.MOT (t = 4.30; *p* < 0.001), FIM.COG (t = −2,55; *p* = 0.014), FIM.TOT (t = 6.17; *p* < 0.001), ADL (t = 4.97; *p* < 0.001), IADL (t = 4.64; *p* < 0.001), and EQ5D.VAS (t = −2.69; *p* = 0.010). In particular, the effect of the experimental treatment involved an improvement in all patients’ test scores, as well as for the control group (Figure 2). However, lowest *p*-values show a larger improvement in the EG, especially in the MOCA, BDI, ADL, and IADL, as shown in Table 4. Finally, we observed a significant change from T0 to T1 in the proportions of the EQ5D.UA (*p* = 0.02) in the CG and the EQ5D.SC (*p* = 0.01), EQ5D.PD (*p* = 0.02), and EQ5D.AD (*p* = 0.01) in the EG.

## 4. Discussion

The purpose of this study was to assess the efficacy of HA training in enhancing personal and social autonomies in SCI patients, with a consequent improvement in cognitive functioning and anxiety–depressive symptoms compared to traditional training. To our knowledge, this is the first study using HA as a rehabilitation tool in patients with SCI. Our study shows that HA training could be effective in improving ADL, social functioning, perceived QoL, life skills independence, and functional recovery in patients with SCI (Table 3). Our findings demonstrate that both the traditional treatment and HA treatment led to statistically significant improvements, indicating that both methods are effective. However, the EG and CG groups differed at follow-up, with the experimental treatment demonstrating a substantial improvement particularly in the EG group, and especially in global cognitive functioning (MOCA = *p*-value < 0.001), depressive symptoms (BDI = *p*-value < 0.001), and activities of daily living (ADL = *p*-value < 0.001; IADL = *p*-value < 0.001), as shown in Table 4.

Over a period of two years, this study focused on implementing a rehabilitative approach that promotes autonomy through the use of HA. To analyze the results, we used ANCOVA to determine whether the type of treatment affected the clinical outcome and the Wilcoxon signed-rank test for inter-group analysis. The findings showed that HA led to an improvement in cognitive functioning, mood disorders, and ADL. This approach holds promise for more effective management of SCI disorders by making use of a patient’s residual resources. Our study highlights the importance of using innovative technologies not only to improve cognitive functions, as previous studies have shown [37,38], but also to develop strategies for coping with new challenges.

Currently, there are no rehabilitative therapies available in an automated environment to improve the residual abilities of SCI patients. After being discharged from the hospital, these patients must reintegrate into their homes. During this phase, patients may suffer from depression due to their medical condition [19] and anxiety symptoms [20] as they confront architectural barriers that were not an issue before their injury. This increases their sense of frustration, social isolation, and inability to function independently. Providing an automated environment that promotes patient autonomy can help alleviate these feelings. By training in a protected rehabilitative context, patients can acquire new skills that can be applied to their daily lives outside of the hospital. In an ecological context, patients experience themselves in a different clinical condition, which allows them to change their point of view about their ability to adapt. According to Bhattarai et al., psychological interventions aimed at strengthening self-efficacy in performing daily activities can improve functional independence, leading to a better quality of life [45]. Our findings suggest that a rehabilitative program based on the improvement of ADL and IADL using HA provides evidence that domotic technologies enhance global cognitive functions, psychological well-being, and QoL compared to conventional treatment (Table 4). Other studies have shown that motor rehabilitation, focused on the upper and lower limbs, improves independence and can potentially benefit psychological status and QoL [46,47]. Our study introduced technological innovation to rehabilitate ADL in a safe environment. This helped with training patients and facilitated their return home. These positive outcomes could have a beneficial effect on patients by reducing their isolation and ease the economic and psychological burden on caregivers and society, potentially improving their QoL.

However, several limitations should be acknowledged to ensure a comprehensive understanding of the findings. The relatively small sample size and the selection criteria for the participants may limit the generalizability of the results to the broader SCI population. Moreover, the study’s duration may not have been sufficient to capture the long-term effects of HA on cognitive functioning, mood disorders, and activities of daily living. Additionally, the method used in the study may be defined as not fully objective from a statistical point of view since patients at the end of the rehabilitation period returned home and did not undergo a follow-up period of monitoring to track any progress or deterioration in their natural environment. Furthermore, while the study included a control group receiving conventional rehabilitation, the nature of the conventional treatment and potential biases in the outcome assessment warrant consideration. Future research should address these limitations and explore environmental factors, long-term sustainability, and cost-effectiveness to provide a more robust evidence base for incorporating HA technology into SCI rehabilitation practices.

## 5. Conclusions

People with SCI make up only 0.1% of the world population. However, SCI can cause immense trauma and difficulties for those affected. Unfortunately, there is no therapy available that can repair damaged nervous tissue. As a result, people with SCI may experience severe limitations to their autonomy and require extensive care [48]. To ensure a good quality of life (QoL), it is critical to provide people with SCI with the right to access proper treatments and the ability to define their life plan and remove any barriers that hinder their social inclusion and participation in various aspects of life.

This interpretative key suggests that disability is the result of the interaction between an individual’s characteristics and their health conditions, as well as the characteristics of the physical and social environment they live in. Over time, there has been a gradual increase in the life expectancy of these patients, leading to new challenges, such as improving their QoL, managing chronicity and aging, and paying greater attention to long-term consequences. Therefore, we believe that using assistive technology, such as HA, in conjunction with clinical practice could be promising for improving the social and cognitive functioning of SCI patients, with positive effects on their QoL. Future research should confirm these results in the long term with major statistical support. This automated environment could promote safe reintegration and optimal recovery of ADL.

## Figures and Tables

**Table 1:** Sociodemographic description of the sample.

	All	EG	CG	*p*-Value
Participants	50	25 (50.0)	25 (50.0)	-
Male	29 (58.0)	16 (64.0)	13 (52.0)	0.57
Age (years)	50.2 ± 15.2	44.6 ± 12.5	55.9 ± 15.7	0.01 *
Education (years)	11.8 ± 3.2	13.0 ± 3.2	10.6 ± 2.7	0.01 *
Spinal Injury Disability (AIS)				
AIS-A patients	24 (48.0)	12 (48.0)	12 (48.0)	0.99 *
AIS-B patients	26 (52.0)	13 (52.0)	13 (52.0)	

Experimental group (EG); control group (CG). Continuous variables were expressed as means ± standard deviation, with categorical variables as frequencies (percentages) * *p* value = < 0.05.

**Table 2:** Examples of activities performed using both traditional methods and HA systems.

Daily Living Activities	Home Automation (HA) Systems	Conventional Methods
**Dishwashing**	A nifty lifting system has been installed in the kitchen cabinets to make them adjustable in height, which is especially useful for wheelchair users. This system has a unique function that not only lowers the cabinet to a height accessible to wheelchair users but also moves the cabinet forward from the wall, bringing it even closer to the user. This way, the user can easily store dishes after washing them, making the entire process more convenient.	In the traditional approach, the therapist would assist the patient in placing the dishes on the shelves. Alternatively, the patient would have to use their own strength to raise themselves up from the chair and store the dishes themselves.
**Oral hygiene**	A brush with a gentle design was used by users to promote circulation in the gums through its massaging action. This type of brush was particularly useful for those with limited mobility. With its compact head and patented bristles, it prevents gagging while brushing the back teeth and eliminates any harsh poking that can occur with straight bristled brushes.	Users were provided with a hand brace to help them grip their toothbrush or were assisted by their therapist to hold onto the handle.
**Cooking**	Users have access to a spacious worktop that comes equipped with a variety of tools for slicing, grating, cleaning, and drying food, as well as for opening cans, jars, and bottles. The cutting board is anchored to the table or kitchen with four suction cups at the base, ensuring stability during food preparation. The raised edge and food stops enable food to be secured in place while being prepared for meals.	Users would have to rely on the assistance of a therapist to help them perform each task involved in food preparation.
**Eating**	Users were able to enjoy the benefits of adjustable cutlery, thanks to a flexible section that eliminates the need for wrist movements when bringing food to the mouth. The grip is comfortable and safe to hold, with a strap closure that can be adjusted to suit the user’s needs. The width of the grip allows for a firm grasp of the cutlery in the palm of the hand.	Users had to rely on the help of a therapist to bring food to their mouth.

**Table 3:** ANCOVA results for each covariance model on clinical assessment.

Clinical Assessment	Group Coefficient				Adjusted R^2^
	Estimate	Std. Error	t Value	*p* Value	
BDI	−4.17	0.60	−6.93	**<0.001**	0.79
HRSA	−1.30	0.32	−4.03	**<0.001**	0.85
SF12.MH	1.30	0.64	2.04	**0.047**	0.18
SF12.PH	0.88	0.30	2.96	**0.005**	0.67
SF12.TOT	2.42	0.62	3.88	**<0.001**	0.55
FIM.MOT	3.14	0.73	4.30	**<0.001**	0.69
FIM.COG	−0.29	0.11	−2.55	**0.014**	0.69
FIM.TOT	4.76	0.77	6.17	**<0.001**	0.74
ADL	0.45	0.09	4.97	**<0.001**	0.59
IADL	0.54	0.12	4.64	**<0.001**	0.54
EQ5D.VAS	−0.26	0.10	−2.69	**0.010**	0.10

Significant differences between treatment effects are in bold. BDI = Beck Depression Inventory; HRSA = Hamilton Anxiety Rating Scale; SF12.MH = 12-Item Short-Form Survey Mental Health Score; SF12.PH = 12-Item Short-Form Survey Physical Score; SF12.TOT = 12-Item Short-Form Survey Total.

**Table 4:** Statistical comparisons of clinical scores between baseline (T0) and follow-up (T1), for both experimental and control groups.

Clinical Assessment	Experimental Group		*p*-Value	Control Group		*p*-Value
	T0	T1		T0	T1	
MOCA	23.0 (21.0–25.0)	26.0 (25.0–27.0)	**<0.001**	22.0 (19.0–25.0)	24.0 (19.0–26.0)	**0.006**
BDI	13.0 (8.0–16.0)	5.0 (2.0–10.0)	**<0.001**	15.0 (10.0–19.0)	14.0 (10.0–18.0)	**0.038**
HRSA	10.0 (6.0–15.0)	7.0 (5.0–10)	**<0.001**	12.0 (6.0–15.0)	10.0 (6.0–12.0)	**0.001**
SF12.MH	18.0 (16.0–22.0)	23.0 (21.0–25.0)	**<0.001**	18.0 (17.0–22.0)	21.0 (19.0–24.0)	**<0.001**
SF12.PH	12.0 (11.0–15.0)	18.0 (15.0–18.0)	**<0.001**	12.0 (11.0–15.0)	15.0 (15.0–17.0)	**0.004**
SF12.TOT	28.0 (21.0–30.0)	36.0 (33.0–39.0)	**<0.001**	29.0 (22.0–30.0)	32.0 (31.0–35.0)	**<0.001**
FIM.MOT	65.0 (58.0–68.0)	78.0 (71.0–82.0)	**<0.001**	63.0 (58.0–68.0)	72.0 (69.0–75.0)	**<0.001**
FIM.COG	31.0 (30.0–33.0)	34.0 (33.0–35.0)	**<0.001**	31.0 (30.0–32.0)	32.0 (31.0–33.0)	**<0.001**
FIM.TOT	95.0 (88.0–100.0)	113.0 (103.0–115.0)	**<0.001**	94.0 (88.0–100.0)	102.0 (97.0–108.0)	**<0.001**
ADL	5.0 (4.0–5.0)	6.0 (5.0–6.0)	**<0.001**	5.0 (4.0–5.0)	5.0 (5.0–5.0)	**0024**
IADL	5.0 (5.0–6.0)	6.0 (6.0–7.0)	**<0.001**	5.0 (5.0–6.0)	5.0 (5.0–6.0)	**0.008**
EQ5D.VAS	51.0 (50.0–60.0)	60.0 (60.0–70.0)	**<0.001**	55.0 (50.0–60.0)	57.0 (55.0–65.0)	**<0.001**

Scores are medians (first–third quartile); significant differences between treatment effects are in bold. MOCA = Montreal Cognitive Assessment; BDI= Beck Depression Inventory; HRSA = Hamilton Anxiety Rating Scale; SF12.MH = 12-Item Short-Form Survey Mental Health Score; SF12.PH = 12-Item Short-Form Survey Physical Score; SF12.TOT = 12-Item Short-Form Survey Total Score; FIM.MOT = Functional Independence Measure Locomotion Score; FIM.COG = Functional Independence Measure Social Cognition Score; FIM.TOT = Functional Independence Measure Total Score; ADL = Activities Of Daily Living; IADL = Instrumental ADL; EQ5D.VAS = EQ-5D = EuroQol-5D Visual Analogue Scale.

## Data Availability

Data are available from the corresponding author.
